# Examining the Impact of an Adapted Professional Development Model for the Advancing Social-Communication and Play (ASAP) Intervention

**DOI:** 10.3390/bs16081363

**Published:** 2026-08-09

**Authors:** Jessica R. Steinbrenner, Stephanie Reszka, Laura J. Kuhn, Rebecca Dees, Rebecca Parkin, Linda R. Watson

**Affiliations:** 1 Department of Health Sciences, University of North Carolina at Chapel Hill, Chapel Hill, NC 27599-7190, USA; stephanie.reszka@unc.edu (S.R.); rebecca_parkin@med.unc.edu (R.P.); linda_watson@med.unc.edu (L.R.W.); 2 Frank Porter Graham Child Development Institute, University of North Carolina at Chapel Hill, Chapel Hill, NC 27599-8180, USA; kuhn@unc.edu (L.J.K.); becky_dees@med.unc.edu (R.D.)

**Keywords:** autism, social-communication, play, preschool, school-based intervention, implementation, fidelity

## Abstract

Given the increasing prevalence of autism among school-aged children, preschool classrooms need evidence-based interventions to address students’ social-communication needs. However, the complex school context presents unique challenges to maintaining a high level of intervention fidelity. We have engaged in an iterative development and testing process of professional development (PD) supports for the Advancing Social-Communication and Play (ASAP) intervention to create a PD model that supports preschool teams in their uptake and intervention fidelity. This pilot cluster randomized control trial included 12 classrooms (42 educators, 43 autistic preschoolers) and compared classrooms that received ASAP with adapted PD supports (ASAP-PD) to ASAP with materials only (ASAP-MO). The PD model supported gains in educators’ knowledge of ASAP content and reported use of ASAP procedures when compared to ASAP-MO classrooms; however, there were no group differences in fidelity of implementation during classroom observations, and implementation varied widely across classrooms. There were also no group differences in primary student outcomes, although parents in the ASAP-PD group reported significant changes in child skills from pre- to post-test. Implications for practice and future directions for research are discussed with a focus on the importance of implementation supports to address common challenges in schools.

## 1. Introduction

According to the Autism and Developmental Disabilities Monitoring Network, the prevalence of autism continues to rise in the United States, with current estimates of 1 in 34 children receiving an autism diagnosis by age 4 and 1 in 31 children receiving a diagnosis of autism by age 8 (based on comprehensive evaluations, special education eligibility, or classification; Shaw et al., 2025). These rising prevalence numbers mean that schools are serving large numbers of autistic students. In the 2023 school year, over 100,000 3–5-year-old children with an educational label of autism were served through Part B IDEA services across the United States, representing 17% of students receiving Part B services in the preschool age group (Office of Special Education, 2025). Given that many of these students receive services in public school settings, the development and use of evidence-based practices (EBPs) that can be implemented in preschool classrooms is critical for supporting the needs of these young autistic children.

Although educators are often eager to implement EBPs in classroom settings, the fidelity of implementation in school-based settings is quite variable (Hugh et al., 2021). This variability is perhaps unsurprising given some of the challenges in public school settings, including teacher turnover and shortages in special education (Gilmour et al., 2025; Learning Policy Institute, 2025), increasing reliance on paraeducators (U.S. Department of Education, Office of Special Education and Rehabilitative Services, 2024) paired with limited training opportunities for this group (Sobeck & Robertson, 2019; Tagavi et al., 2025), and high caseloads/workloads for teachers and related service providers (American Speech-Language-Hearing Association, 2024; Dewey et al., 2017). Collectively, these challenges mean that classroom teams are often under-prepared and under-resourced to provide high-quality EBPs in increasingly complex school settings. Indeed, many educators report gaps in knowledge or experience related to autism characteristics and autism-specific EBPs (Hugh et al., 2024; Petersson-Bloom & Hansson, 2025; Tagavi et al., 2025) and low confidence around EBP implementation related to addressing critical needs for autistic students (Brock et al., 2014; Layden et al., 2022).

EBPs that address social and communication skills are of particular importance for supporting autistic preschoolers or developmentally delayed preschoolers who may go on to receive a diagnosis of autism since social-communication is one of two core areas of deficit or difference for autistic individuals (American Psychiatric Association, 2013) and one of the first areas of concern even prior to diagnosis (Leach et al., 2025; Zablotsky et al., 2017). Although assessment and goal development related to social-communication skills are often led by speech-language pathologists, these skills are critical across nearly all contexts. Thus, it is important for all members of educational teams to be knowledgeable and skilled in supporting social-communication. As such, interventions directly addressing social-communication skills for young autistic children that also incorporate a team-based approach to addressing these skills are critical in complex environments, such as school-based settings.

### 1.1. History of ASAP

The Advancing Social-Communication and Play (ASAP) intervention (Watson et al., 2011) was iteratively developed as a school-based adaptation of a joint attention and symbolic play (JASP) intervention initially developed by Kasari et al. (2006, 2008). JASP, and subsequently ASAP, were designed to address pivotal developmental skills which are highly predictive of children’s long-term developmental and functional outcomes (Laakso et al., 1999; Mundy & Crowson, 1997). ASAP is a fully manualized, team-implemented intervention to address core areas of need for autistic preschoolers and was iteratively designed in partnership with public preschool programs to be layered with the curricula and EBPs that preschool teams are already using (Dykstra Steinbrenner et al., 2015). It generally aligns with naturalistic, developmental behavioral intervention principles but can be used broadly across a variety of classroom models. ASAP addresses two content areas (social-communication and play), across two contexts (focused and generalized practice), and utilizes a cyclical process (assess, plan, teach) to support teams in addressing these core areas of need with their autistic preschool students.

Early pilot studies suggested that aspects of ASAP were successful in promoting social-communication and play skills (Dykstra et al., 2012; Dykstra Steinbrenner et al., 2015; Watson et al., 2009). A larger, multi-site cluster-randomized controlled efficacy study with 161 students (Boyd et al., 2018) demonstrated that ASAP had moderate effects on children’s total classroom engagement (d = 0.50), with a notable but nonsignificant effect on coordinated joint engagement (d = 0.39), which is closely aligned with the joint attention goals in ASAP. However, there were neither time nor group effects in generalized social-communication or play skills (i.e., when assessed outside of the classroom by naïve examiners). This pattern of findings contrasted with outcomes in two preliminary studies from the ASAP development project, in which the ASAP group showed improvements in generalized social-communication and play skills.

Notably, there were decreasing levels of fidelity of implementation (FOI) as the intervention progressed further into authentic classroom-based settings and larger studies. The FOI was 92% for research team implementers (i.e., research assistants in a speech-language pathology graduate program), 81% FOI for educators during two studies in the ASAP development project, and 56% FOI for educators in the multi-site efficacy trial (Reszka et al., 2019). Difficulties in the attainment of sufficient FOI during the multi-site efficacy study highlight the need to give the same level of attention to developing a feasible, effective, and sustainable professional development (PD) approach to support educators in implementing innovations such as ASAP as was given to developing the intervention itself (Barry et al., 2020).

### 1.2. Iterative Adaptations to ASAP Professional Development (ASAP-PD)

Research team members across previous ASAP studies highlighted challenges such as inconsistency in perceived buy-in across teams and team members, delays in initiation of the ASAP assessments and intervention after initial training, insufficient density of targeted skills and dosage of focused practice, and inconsistent use of EBPs to address ASAP skills. To address challenges with FOI found in previous ASAP research, this study formed an interdisciplinary team to iteratively adapt and pilot test the ASAP-PD model. We used a multi-phased study using mixed methods approaches guided by an implementation science framework using the Normalization Process Theory (NPT; May et al., 2009), which has been widely used in health care settings (May et al., 2018) and adapted for education settings (Wood, 2017). The NPT framework includes four factors that impact implementation: coherence (the sense-making work of understanding an innovation), cognitive participation (the relationship-building work of team building and organizing the work for the innovation), collective action (the operational work of learning about and executing the innovation), and reflective monitoring (the appraisal work of assessing success and adapting the innovation). The NPT was selected to address several of the challenges experienced in previous studies by supporting buy-in and identifying fit of ASAP within existing classroom practices (coherence), enhancing supports for organizing the work across team members (cognitive participation), embedding ASAP in everyday classroom routines (collective action), and building more explicit opportunities for self- and team-reflection throughout the training and coaching process (reflective monitoring).

The overarching study included three primary phases. Phase 1 was a combination of concurrent and sequential activities including (a) several activities using qualitative methods (e.g., interviews, focus groups, artifact reviews) that used the Rapid Assessment Process (Beebe, 2001) to code data based on the NPT framework, (b) a survey of preschool team members, (c) a systematic review of team-based PD models in educational settings, and (d) regular meetings with an advisory board. Based on data from these activities, ASAP-PD materials were adapted, reviewed, and further revised. Phase 2 used design experimentation to iteratively test the updated ASAP-PD supports, although it required a pivot to test with interdisciplinary groups of graduate students based on challenges with recruitment of public school classrooms just after the COVID-19 pandemic. Phase 3 was the culminating pilot study of the adapted ASAP-PD program. It included a pilot cluster randomized control trial (CRCT) to compare classrooms that received ASAP with adapted PD supports (ASAP-PD) to ASAP with materials only (ASAP-MO), which is the focus of this paper. The team also conducted a parallel Implementation and Procedural Evaluation (IPE) to provide a richer understanding of implementation, though reporting on the full IPE is beyond the scope of the current paper.

The current study reports on the outcomes of the CRCT and includes supplemental quantitative data from the IPE. The primary aims of the current study are below.

Aim 1: Examine the impact of ASAP-PD compared to ASAP-MO on classroom teams’ FOI.

Aim 2: Examine the impact of ASAP-PD compared to ASAP-MO on student engagement and social-communication.

Aim 3: Explore practical factors related to implementation and student outcomes of ASAP-PD and ASAP-MO.

We hypothesized that classrooms receiving the NPT-informed, enhanced ASAP-PD would have a greater understanding of ASAP procedures (i.e., knowledge) and how ASAP fit within their existing classroom context, which would lead to improved ASAP implementation in the classroom (i.e., quality, dosage) compared to classrooms receiving ASAP-MO. Although our more proximal outcome was for educators (e.g., understanding and implementation of ASAP), we also hypothesized that higher ASAP knowledge and implementation would ultimately lead to improved student outcomes in engagement and social communication as a distal outcome.

## 2. Materials and Methods

The pilot CRCT comparing ASAP-PD and ASAP-MO took place in two school districts in the southeastern United States and included autistic preschoolers and educational teams supporting these students in public school settings. The study was approved by the University of North Carolina at Chapel Hill Institutional Review Board (IRB# 20-1507), and the study procedures included participant recruitment, pre-assessments, randomization, intervention, and post-assessment, and are described in further detail below.

### 2.1. Participants

**Figure 1:**
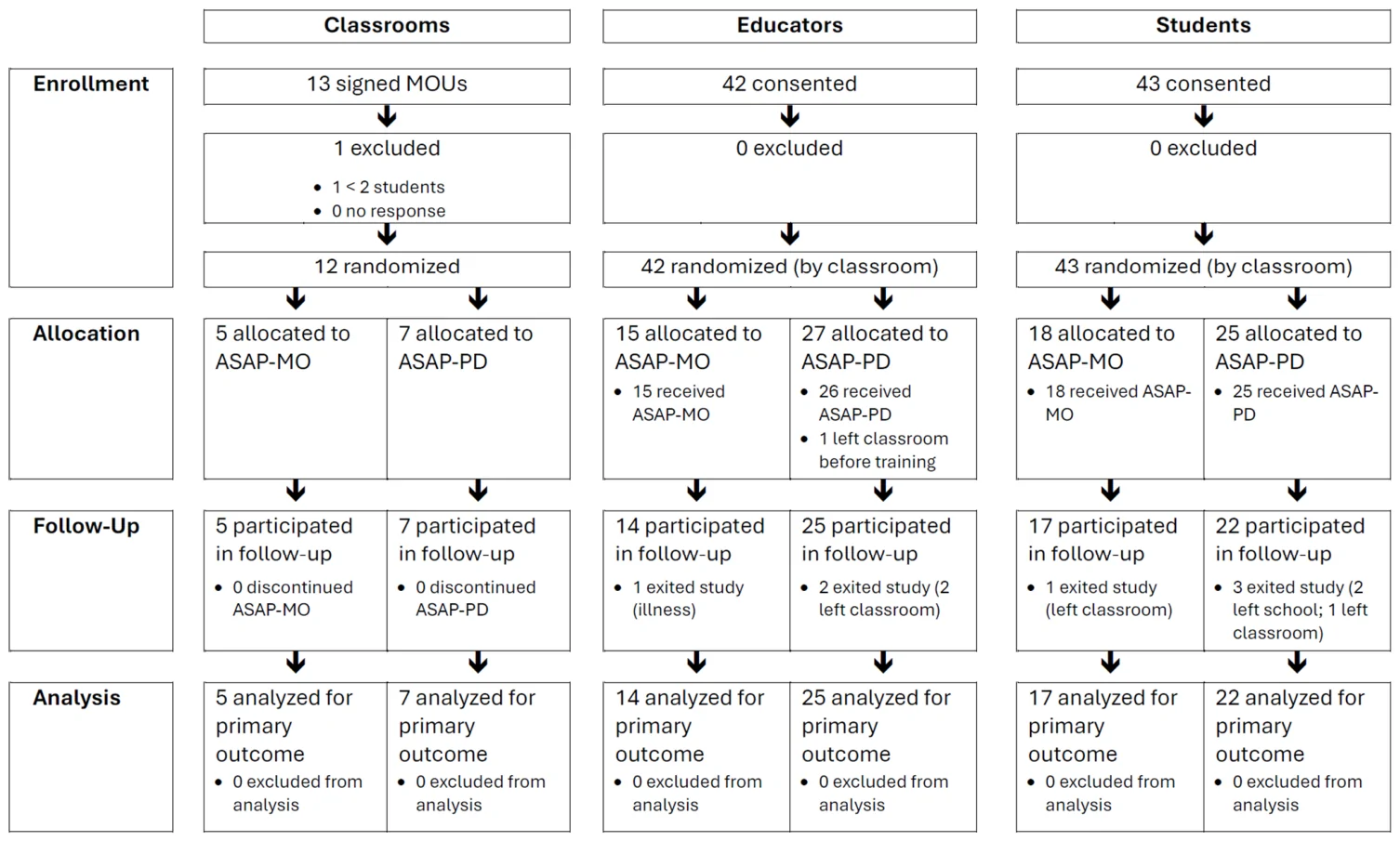
Adapted CONSORT Diagram. The adapted CONSORT diagram shows classroom, educator, and student participants for the current study in separate sections, with each column further divided by group assignment (ASAP-MO and ASAP-PD). The arrows indicate progression through study phases that include enrollment, allocation, follow-up, and analyses.

The research team sought approval from seven school districts and ultimately worked with two districts. Once we received approval, we worked with district staff to identify preschool classroom teams who were serving at least two autistic students with social-communication and/or play needs, with a goal of enrolling 20 preschool teams in the study. ASAP is a team-based intervention, so we aimed to recruit and enroll educational teams that included at least three members: a lead teacher, a teaching assistant, and a related service provider (RSP) such as a speech-language pathologist (SLP) or occupational therapist (OT). After an initial interest meeting, the lead teacher and a school administrator (principal or assistant principal) reviewed and signed a memorandum of understanding, and interested preschool team members completed study consents. Then, lead teachers distributed parent permission packets to families of eligible autistic students. Classrooms were enrolled in the study if they had at least two educational team members and two autistic students with consent to participate in the study. Based on recruitment challenges, there were ultimately 12 classrooms, 42 educational team members, and 43 students enrolled in the study across two cohorts during the 2023–2024 and 2024–2025 school years. See Figure 1 for a CONSORT diagram.

### 2.2. Educational Team Members

Inclusion criteria for educators were that they were working as part of a public preschool classroom team that supported autistic students. Lead teachers and RSPs had to be certified or licensed for their role in the state. Additionally, lead teachers had to have at least one year of teaching experience with autistic students at any age (i.e., not a first-year Exceptional Children teacher).

Across the 12 classroom teams, 42 educators enrolled in the study, though 1 left very early in the study. One team was unable to recruit a related service provider, but the classroom team was still interested in participating and enrolled in the study. The 41 educators who completed demographic forms included 12 lead teachers, 18 teaching assistants, and 11 related service providers. See Table 1 for additional demographic information about educators.

### 2.3. Students

Inclusion criteria for preschool students were that they had an educational and/or medical diagnosis of autism or a suspected diagnosis of autism and received Individualized Education Plan (IEP) services through an enrolled classroom. Students were excluded from the study if they had an uncontrolled seizure disorder, traumatic brain injury, and/or severe, uncorrected visual or hearing impairment. There were no exclusion criteria based on languages spoken at home, though parent permission forms and other forms completed by families were only available in English and Spanish.

Across the 12 classrooms, 43 autistic preschool students were enrolled in the study, with a range of 2 to 5 per classroom. Across groups, 65% (n = 28) of the students were from a racial or ethnic group that has been historically marginalized in the United States, and 30% (n = 13) of students’ families had household incomes below USD 40,000. The average age of the children in the study was slightly over 4 years, and 70% (n = 30) were male. An analysis of participant characteristics showed no differences between the control and treatment groups in child age or baseline social-communication or joint attention skills (see Supplemental Table S1). Table 2 has additional demographic information about student participants by group.

At enrollment, students completed the Visual Reception and Expressive Language subscales of the Mullen Scales of Early Learning (MSEL; Mullen, 1995). Due to floor effects on standardized scores, Mullen raw scores were used for all analyses. There were significant group differences in MSEL Visual Reception scores, with children in the ASAP-PD group having significantly lower raw scores (ASAP-MO = 27.35, age equivalent 25 months; ASAP-PD = 22.88, age equivalent ~20 months, *p* = 0.05). Finally, research staff completed the Childhood Autism Rating Scale (CARS-2; Schopler et al., 2010), an observed measure of autism symptom severity of students based on videotaped interactions during assessment administration. Two research staff members scored the CARS-2 independently based on viewing video-recorded assessments and report measures gathered from caregivers and teachers. The team members resolved any item-level disagreements through consensus. Student scores ranged from 29 to 49, with a mean of 39.6 (SD = 5.0). A total of 2 students’ scores fell in the ‘no to minimal symptoms’ range (though at or very near the cut-off score), 10 students’ scores fell in the mild to moderate symptoms range, and the remaining 31 students’ scores fell in the severe symptoms range.

Over the course of the school year, 4 children withdrew from the study (due to changing classrooms (n = 2) or moving to another school (n = 2). An attrition analysis indicated there were no group differences in personal and family demographic characteristics or in baseline assessments for children who withdrew from the study (see Supplemental Table S2).

### 2.4. Intervention and Randomization

The aim of this study was to examine a PD model for the ASAP intervention; thus, all classrooms were instructed to provide ASAP to enrolled students, received the ASAP intervention materials, and were randomized to receive different levels of PD supports. Given the small sample size of this pilot study, we matched based on classroom and school characteristics prior to randomization to increase the likelihood of having similar characteristics across groups. Classrooms were matched within districts, with the first priority of matching based on classroom schedule (full day versus half day) and then on the percentage of students receiving free and reduced-price lunch at the school. The classroom IDs of matched pairs were then provided to the statistician (naïve to all school information) and randomized to ASAP-PD and ASAP-MO in SAS v9.4 using prox surveyselect.

### 2.5. ASAP Materials and Professional Development Procedures

Both groups (ASAP-PD and ASAP-MO) received two hard copies of the ASAP manuals (a collection of three books) and access to the publicly available ASAP website. The first book of the manual provides the rationale, description, and procedures related to the ASAP skill hierarchies, assessments, and intervention. The second book consists primarily of activity ideas for focused practice and generalized practice. The third book is a parent handbook to support collaboration with parents. The ASAP website (www.med.unc.edu/healthsciences/asap/, accessed on 1 June 2026) includes the manuals in PDF format, supplemental resources (e.g., tip sheets, additional family collaboration supports), and a 1 h training video.

The ASAP-PD group additionally received training and coaching to support their implementation across the school year. The training included four modules: ASAP Orientation, ASAP Play, ASAP Social-Communication, and ASAP Procedures. Each module included interactive, web-based training that was completed independently by all team members (~20–40 min in length) and guided team training (~30–45 min in length) led by an ASAP coach (i.e., research team member). Following the initial training, teams participated in ongoing coaching. The ASAP coach completed two observations per month in which they completed fidelity checklists on team members implementing ASAP in the classroom, and one team meeting per month that included team discussion about successes and challenges, positive and constructive feedback based on the fidelity checklist, and team reflection, goal-setting, planning, and problem-solving. The coaching process was led by a research team member but was collaborative in nature and guided by input from classroom team members (e.g., selecting observation times, choosing team goals, identifying action steps). Key changes to the ASAP-PD included (a) the modular training format, which was designed to be more flexible and feasible with team members’ schedules and availability as well as provide homework (e.g., conduct ASAP assessment) between training sessions to reduce barriers to initiating ASAP, (b) increased discussion and addition of tools to support allocation of ASAP work among team members, (c) addition of individual and team reflection questions and discussions in the training modules and coaching meetings to support educator buy-in, as well as earlier identification of challenges and solutions, and (d) update and creation of tools related to ASAP implementation (e.g., educator tip sheets, ASAP materials guide) to provide tangible problem-solving supports in coaching meetings.

### 2.6. Measures

This study assessed team implementation of ASAP (Aim 1, proximal outcomes), student engagement and social-communication (Aim 2, distal outcomes), and metrics of social validity and other practical factors related to implementation (Aim 3). All measures are described below.

#### 2.6.1. Implementation Outcome Measures

The fidelity of implementation (FOI) measure was designed to document general knowledge of ASAP content, use of the prescribed assess–plan–teach cycle, as well as educator teaming and identified facilitators/barriers to ASAP implementation. There were three components to the ASAP fidelity measure. (1) Interview: interview with lead teacher, conducted by a research team member, transcribed, and coded by another research team member who was naïve to group assignment; (2) Artifact Review: scored based on materials submitted by the classroom team to the research staff; and (3) Observation: live classroom observation of approximately 60–90 min completed by two raters (at least one naïve to group assignment) with independent followed by consensus scoring. There are three key features of ASAP fidelity: ASAP Knowledge and Procedures, ASAP Classroom Implementation, and General Classroom Practices (see Table 3 for more details). Items across all measures were operationalized to be scored on a 0 (no or little implementation) to 2 (strong or full implementation) scale.

**ASAP Knowledge and Procedures.** The scores for ASAP Knowledge and Procedures combine data coded from the educator interviews related to adherence to the prescribed ASAP procedures of goal selection, assessment process, and teaming. Interviews were completed at a mid-point and post-test for Cohort 1 classrooms; however, due to delayed recruitment and subsequent time constraints, Cohort 2 interviews were only completed at post-test, thus mid-point interview data are not available for Cohort 2. Data at post-test were consistently gathered at the end of the classrooms’ participation in the intervention, allowing comparison of these aspects of classroom fidelity across all classrooms only at the beginning and end of implementation, but not at the mid-point. The ASAP Knowledge and Procedures data exhibited strong internal reliability (α = 0.94 at mid-point and α = 0.87 at post-test).

**ASAP Classroom Implementation**. The classroom observation focused on the implementation of ASAP in the classroom setting across both the focused and generalized practice settings in which ASAP was designed to be implemented. Items captured the preparation for the activity, implementation, educator monitoring of child progress toward goals, and child responsiveness during the activity. Items were the same for the focused and generalized contexts, and items were only scored if team members were observed to be addressing an ASAP social-communication or play goal in a context. There were few classrooms in which the generalized practice addressed an ASAP goal during scheduled observations, so the ASAP Classroom Implementation reported only includes the focused practice observation scores. The ASAP Classroom Implementation for focused practice and generalized practice had strong internal reliability within our sample (α = 0.90 and α = 0.95, respectively).

**General Classroom Practices.** The ASAP-PD General Classroom Practices documents the use of EBPs and facilitating features that may support the use of ASAP in the classroom. Items were drawn from the Autism Program Environment Rating Scale—Preschool/Elementary (APERS-PE; Odom et al., 2024), which has been used successfully in other school-based studies (e.g., Odom et al., 2018, 2022) including preschool classrooms (Bejnö et al., 2023). EBPs include items related to the use of reinforcers, feedback, and prompting, as well as visual supports and predictable routines that are considered foundational evidence-based practices for use in classrooms serving autistic preschool-aged children (Hume et al., 2021). Facilitating features include items related to the broader classroom environment (e.g., safety, organization) as well as teacher/staff interactions with children. The ASAP General Classroom Practices exhibited good internal reliability at pretest (α = 0.78) and post-test (α = 0.80).

#### 2.6.2. Student Outcome Measures

**Engagement.** The Behavioral Observation of Students in Schools—Early Education (BOSS-EE; Alperin et al., 2023) is a momentary time-sampling measure of child engagement designed for use in classroom settings. Engagement states include active (verbal and/or motor engagement), passive (demonstrated interest, gaze, orientation), and non-engagement (interfering and self-directed behaviors, non-participation). For the purposes of this study, engagement was the total number of 15-s intervals in which the child was coded in active engagement. The BOSS was scored by research staff who were naïve to group assignment, based on mean scores of two 5-min observations in the classroom context, for a total of 10 min per child at pretest and post-test. Inter-rater reliability was completed for 20% of observations and was over 0.80 for both time points.

**Communication outcomes.** Communication outcomes were collected via a video-recorded, semi-structured, play-based interaction completed with the student and a research team member (i.e., unfamiliar adult) who was naïve to group assignment. The play-based interaction combined play activities from two assessments, the Early Communication Inventory—Autism (ECI-A; Nowell et al., 2024) and the Brief Observation of Social Communication Change (BOSCC; Grzadzinski et al., 2016). The semi-structured interaction included two 6-min segments of naturalistic play and four shorter embedded activities designed to elicit joint attention, with a total time of approximately 15–18 min. The video-recorded interactions were coded and scored by trained research team members who were also naïve to group assignment. Inter-rater reliability was collected on at least 25% of observations at pretest and post-test. Reliability was above 0.80 for both the ECI-A and BOSCC scores at both time points.

The ECI-A was coded using the first 6-min segment and four elicitation activities described above. The ECI-A includes three different scores: weighted communication rate, directed communication, and initiation of joint attention. The weighted communication rate is scored by totaling weighting tallies of gestures (1 point), vocalizations (1 point), single words (2 points), and multi-word utterances (3 points) that occur in a 6-min period of house/barn play and then dividing by the number of minutes. Directed communication is rated on a scale of 0 (no communication) to 3 (reciprocal directed communication) for each of the four elicitation activities and then averaged for a mean directed communication score. Initiation of joint attention (IJA) is scored as 0 (not present) or 1 (present) for each of the four elicitation activities and then averaged, resulting in a proportion of IJA. The BOSCC includes scoring on 12 items (each item scored 0–5), with each score based on the frequency and quality of different social-communication behaviors (e.g., eye contact, facial expressions, and vocalizations). The items are scored for each 6-min interaction, summed for each of the two segments, and averaged together for a Total Mean BOSCC score (range of 0–5).

#### 2.6.3. Practical Factor Measures

In addition to primary outcome measures, we also gathered data about educators’ perception of ASAP-PD, educators’ and parents’ perceptions of the ASAP intervention, self-report of ASAP implementation, and educators’ and parents’ perceptions of student outcomes.

To assess perceptions of ASAP-PD, educators in the ASAP-PD group completed a PD-ASAP Training Rating questionnaire (10 items on a 6-point rating scale of strongly disagree to strongly agree) after participating in the training modules and the Coach-Teacher Alliance—Teacher questionnaire (Bradshaw et al., 2009; 30 items on a 5-point rating scale of never to always) at the end of the school year. In order to assess perceptions of ASAP, educators and parents across both groups completed the Intervention Rating Profile (IRP; Witt & Martens, 1983), which yields a single dimension of intervention acceptability. The educator version contains 15 items, while the parent version contains 8 items.

Perceptions of student outcomes were measured using the Video-Anchored Ratings of Skills (VARS) developed as part of a previous ASAP study (Dykstra et al., 2012) and uses a 5-min video of a sex-matched neurotypical child to “anchor” parent and teacher ratings on six questions about the target child’s social-communication and play. Educators (VARS-E) and parents (VARS-P) completed the VARS for each student at pretest and post-test.

Finally, to gather data about teams’ implementation of ASAP, the research team completed the ASAP FOI measures (described above for Aim 1), and educators completed the ASAP Self-Report Survey at the end of the study, which included items about implementation at the team and educator level. On the self-report survey, educators were asked if they implemented ASAP less than expected, as much as expected, or more than expected, and then educators checked boxes of factors that may have influenced their implementation.

### 2.7. Analytic Strategy

The planned analyses addressed the three primary aims: implementation outcomes (Aim 1), student outcomes (Aim 2), and practical factors (Aim 3). Prior to running models, assumptions related to homoscedasticity and independence of errors were evaluated. Residual plots were examined for all independent variables to assess potential violations of assumptions. Visual inspection of residuals did not indicate the need for any data transformations.

Intent-to-treat (ITT) analyses were used to address Aim 1 and tested whether fidelity indicators differed between ASAP-PD and ASAP-MO groups using generalized linear modeling (GLM) to assess classroom faithfulness of intervention implementation. ITT analyses were also used to address Aim 2 and tested whether random assignment to the ASAP-PD condition, regardless of participation, attendance, or quality of implementation, is associated with differential child outcomes in areas of social-communication and engagement. For Aim 2, two-level hierarchical linear modeling (HLM) was used to accommodate the nesting of children within classrooms. For Aim 3, descriptive analyses, paired sample t-tests, and GLM were used to examine data related to practical factors and the ASAP and ASAP-PD interventions.

In all ITT analyses, using Proc Mixed in SAS 9.4, models were specified with a random intercept for classrooms. The models included treatment as the predictor of interest and several covariates. Students’ MSEL Visual Reception score (due to baseline group differences), age at assessment, and pretest scores were included as covariates in models examining child outcomes to reduce variability. Models with covariates were grand mean centered to aid in interpretation.

## 3. Results

### 3.1. Implementation Outcomes (Aim 1)

Results presented in Table 4 indicate treatment differences on some fidelity outcomes. Classrooms assigned to ASAP-PD demonstrated higher scores in the ASAP Knowledge and Procedures measure at both the midpoint and post-test than those in the ASAP-MO group. However, there were no differences in the ASAP Focused Practice measure. The effect sizes (Cohen’s d) for the differences are large across all three implementation outcomes. The differences between the ASAP Generalized Practice and ASAP Artifacts measures were not included in analysis as originally planned because few classrooms had scores for each measure.

### 3.2. Student Outcomes (Aim 2)

Results presented in Table 5 indicate no treatment differences on any of the five student outcomes analyzed with 2-level HLMs that included random intercepts for classrooms and age at testing and pretest scores as covariates.

### 3.3. Practical Factors (Aim 3)

The practical factors examine the social validity of ASAP-PD, ASAP, and student outcomes, as well as factors related to implementation collected as part of the IPE. Table 6 includes data by group for the various social validity measures.

#### 3.3.1. Social Validity of ASAP-PD and ASAP

ASAP-PD training ratings were completed by 19 of 26 school-based implementers in the ASAP-PD group. Scores indicated that educators were satisfied with the ASAP training modules, with a mean rating in the agree-to-strongly agree range. The Coach–Teacher Alliance measure was completed by 22 of 26 educators in the ASAP-PD group. Overall, educators’ scores indicated a high level of alliance with the ASAP coach.

When considering the ASAP intervention, educators rated ASAP as high in acceptability with a mean score of 5.3 (out of 6) across the 15 items of the IRP. The scores were similar between the ASAP-PD and ASAP-MO groups, suggesting that acceptability was not impacted by condition. Parents who completed the abbreviated IRP rated ASAP as relatively high in acceptability with a mean score of 4.98 out of 6 across the eight items. These scores were also similar across conditions.

#### 3.3.2. Social Validity of Student Outcomes

The VARS was completed for students at pre- and post-test by parents and educators (see Table 6). Paired sample t-tests indicated significant changes in targeted skills from pre- to post-test as rated by parents and teachers, respectively, for the full sample (*t* = 2.10, *p* = 0.026 and *t* = 2.07, *p* = 0.046), and for parents for the ASAP-PD group (*t* = 2.13, *p* = 0.031). There were no significant differences for the parents for the ASAP-MO group (*t* = 1.49, *p* = 0.15) or the parents or educators, respectively, for the ASAP-MO group (*t* = 1.22, *p* = 0.13 and *t* = 1.46, *p* = 0.08).

#### 3.3.3. Implementation of ASAP

**Figure 2:**
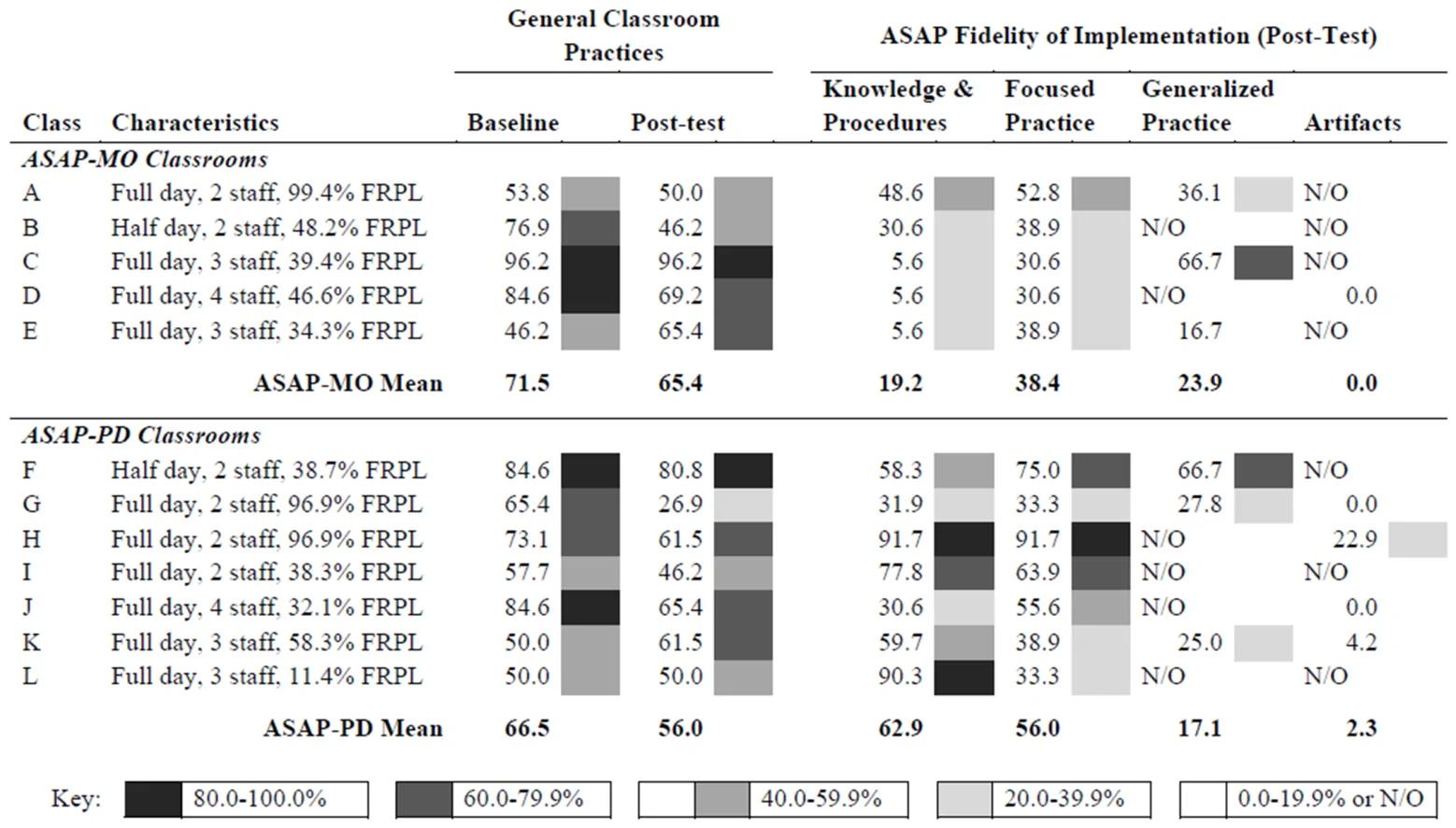
Patterns in Classroom-Level General Practices and Fidelity. Graphic reports characteristics of classroom (full versus half day, number of staff regularly in classroom (i.e., teachers and teaching assistants), and % of students at school level receiving free and reduced price lunch) and then provides percentages with visual key for levels of fidelity related to general classroom practices and Advancing Social-Communication and Play (ASAP) implementation. Note: ASAP = Advancing Social-Communication and Play intervention; ASAP-MO = received ASAP materials only; ASAP-PD = received ASAP professional development model, FRPL = free and reduced-price lunch, N/O = not observed (counted as 0 for means).

Implementation was descriptively examined at the classroom level by dividing classrooms’ FOI scores by 2 (highest possible points) to generate a metric of percent fidelity. Despite significance differences between groups on some implementation measures (see Aim 1 results), general classroom practices and ASAP implementation were highly variable across classrooms and low overall (see Figure 2). For general classroom practices, there was a wide range of scores at pretest for both ASAP-PD (50–85%) and ASAP-MO (46–96%). Some classrooms maintained similar scores on general classroom practices at post-test, but there were also many classrooms with decreases in scores, with notable drops of over 30% for one classroom from each group. Turning to ASAP FOI, the ASAP Knowledge and Procedures were at or above 75% for three of seven ASAP-PD classrooms and no ASAP-MO classrooms. The ASAP Focused context observations were at or above 75% for two of seven ASAP-PD classrooms and no ASAP-MO classrooms. The ASAP Generalized context observations and ASAP Artifact ratings were not observed in six and seven of the classrooms, respectively (and thus not included in the Aim 1 analyses), and were quite low fidelity when observed.

For educators’ self-report of implementation of ASAP, 39 of 43 educators completed the survey (25 of 26 in the ASAP-PD group and 14 of 14 in the ASAP-MO group). A total of 6 educators reported using ASAP more than expected (12% of ASAP-PD and 21% of ASAP-MO respondents), 21 educators reported using ASAP as much as expected (68% of ASAP-PD and 29% of ASAP-MO respondents), and 12 educators reported using ASAP less than expected (20% of ASAP-PD and 50% of ASAP-MO respondents). Of those who used ASAP less than expected, 9 of the 12 educators indicated school/district factors with new students in the class (n = 6), staffing issues (n = 3), and other meetings (n = 3) selected most frequently. Only 2 of 12 educators indicated student factors as the reason for less implementation, noting challenging behaviors (n = 1) and other (n = 2). A total of 4 of 12 educators indicated personal factors impacted implementation, with all selecting other (n = 4). Notably, both of the two educators selecting “other” student factors wrote in about increasing numbers of students in classes, and two of the four educators selecting “other” personal factors wrote about caseload size as a barrier.

## 4. Discussion

This underpowered pilot study of ASAP-PD compared to ASAP-MO showed modest differences in implementation measures based on educator reports but null findings for observed implementation and student outcomes. Social validity and self-report implementation data from the parallel implementation and procedure evaluation (IPE) study provide additional context for the null findings that can inform how we support preschool educational teams in implementing EBPs that address the social-communication needs of autistic students.

### 4.1. Implementation Outcomes

The results suggested the ASAP-PD model supported strong gains in educators’ knowledge of ASAP skill hierarchies and reported use of ASAP procedures (i.e., assessments, progress monitoring, teaming) compared to educators who were merely provided with ASAP materials. Given that knowledge of the social-communication and play hierarchies and use of ASAP procedures are important components of implementation, this positive result is promising. However, the impact of ASAP-PD was not evident in observed ASAP implementation, as there were not significant differences between groups in ASAP focused practice (i.e., dense practice in individual or small group settings), and ASAP generalized practice activities (i.e., dispersed practice in large group settings) were observed in less than half of ASAP-PD classrooms.

The gap between educators’ reports of ASAP knowledge and use and their observed implementation of ASAP could be for several reasons. It is possible that educators in the ASAP-PD group exhibited a social desirability bias in their responses in the implementation interviews, particularly related to their report of ASAP procedures. It is also possible that there is a delay between knowledge and understanding and growth in skills, and the length of time between the end of training and observation (generally around 4 months) was insufficient to see the differences in skill growth. Another potential reason is that the ASAP-PD was helpful in changing knowledge but less effective in changing practice. Finally, since practice was only measured at post-test due to logistical constraints, it is possible that implementation decreased at the end of the year due to external factors like increasing class sizes and competing demands of end-of-year IEP and transition meetings.

There are several plausible reasons for the lack of differences between groups in the FOI observations. First, the smaller-than-anticipated sample size likely impacted our power to detect more subtle differences, especially when both groups had some exposure to the ASAP materials. Second, the observational ASAP FOI measures were collected on a single day for 60–90 min, so may be subject to measurement error, which again is more of a threat to validity in smaller studies. Finally, it is possible that these scores are accurate reflections, and that the ASAP-PD model is not sufficient for creating change in all classrooms.

Related to the final point, the ranges for the focused practice scores were quite different between groups, with several of the ASAP-PD classrooms performing higher than any classroom in the ASAP-MO group. However, there were also several ASAP-PD classrooms that were well below expected implementation levels (i.e., four classrooms under 60%). One potential reason for these differences could be related to differences in general classroom practices, since ASAP is designed to be overlaid on existing classroom structure and curricula. The use of core EBPs and presence of facilitating features may serve as an important foundation for ASAP implementation. Indeed, in looking at the classroom-level data (see Table 6), it appears that the classrooms that scored higher in the ASAP FOI observations tended to have higher general classroom practices, although this is not uniformly the case.

Notably, several of our FOI components (ASAP generalized practice, ASAP artifacts) were absent in many classrooms, which resulted in not including these components in our planned analyses. The absence of these measures is likely reflective of low implementation, but it also means that we were not able to analyze differences in some aspects of implementation.

Our implementation results are consistent with research in special education and autism intervention. Studies have shown that a comprehensive PD approach (e.g., training and coaching) is important for enacting change (Petersson-Bloom et al., 2023; Snell et al., 2013), but also not always sufficient in the face of significant structural barriers, such as those that exist in complex school-based settings (Mandell et al., 2013; Stahmer et al., 2017, 2025; Suhrheinrich et al., 2021). These results also align with research on intervention implementation and behavior change, which notes the impact of other factors like educator confidence, competing priorities, adaptability, and leadership engagement on intervention implementation in the school context (Chapman & Elbaum, 2021; Gallagher et al., 2023).

In sum, there is some evidence that ASAP-PD resulted in educator-reported change in ASAP implementation, but the changes were not always evidenced in observations. The mean implementation was low overall, and implementation was widely variable between participating classrooms. This suggests that ASAP-PD may be sufficient for some but not all classrooms and that additional implementation supports may be important to support a wide variety of educational teams in implementing ASAP and to mitigate the effects of structure barriers impacting ASAP implementation.

### 4.2. Student Outcomes

The results did not indicate any differences in student outcomes based on group assignment. This mirrored a challenge in the previous large-scale CRCT of ASAP, in which there were not significant differences in communication or play skills between groups, though the previous study did find significant differences in engagement (Boyd et al., 2018). Notably, early intervention programs overall have less marked impacts as programs move from model programs (e.g., in university settings) into more authentic community settings (Nahmias et al., 2019). Unlike the previous ASAP study (Boyd et al., 2018), all classroom teams received at least some exposure to ASAP, as they were all provided with the manual and had access to the website, which may have influenced our ability to detect differences between groups in the more distal measure of student outcomes. It is also important to note that the Mullen Visual Reception scores were significantly different between groups, with students in the ASAP-PD group exhibiting lower skills at baseline. Other research has noted different trajectories for skill development based on developmental or cognitive abilities (Perry et al., 2013; Préfontaine et al., 2023), so that may have impacted results given the small sample size. Finally, and importantly, ASAP implementation was low overall across many classrooms, so likely not sufficient to result in student change.

Related to communication, we utilized recently developed measures that were designed to be sensitive to change (ECI-A and BOSCC) in the current study; however, there was limited growth in the sample as a whole. A post hoc paired-sample t-test indicated that only one of the four communication outcome measures (ECI-A directed communication) showed significant growth from pretest to post-test (*t* = 2.49, *p* = 0.017). There may be several reasons for this. It is possible that the students did not grow in other communication areas (rate, initiation of joint attention, social communication) or that the measures were not sensitive to change for these skill areas in this group of students. Since these were play-based measures with a research staff (i.e., an unfamiliar adult), it is also possible that the differences in child/adult dynamics had an impact on the elicitation of the skills. Of note, only 4 of 39 students had the same play partner for both assessment points, and we had 8 different assessors across time points with only 2 in common at pretest and post-test.

Related to engagement, a post-hoc paired-sample t-test showed no evidence of growth in active engagement (*t* = 0.07, *p* = 0.94), which was coded in two 5-min segments per child. While the research team aimed to capture these segments during similar activities during pre- and post-test (i.e., center time), these short segments may not have been sufficient for creating a stable measure of engagement. Additionally, although not formally documented, the classrooms’ student/teacher ratios increased over the course of the year, which is a unique aspect of preschool special education classrooms as children age into Part B services throughout the school year. Previous research indicates that group size can impact engagement during in-class activities (Dykstra Steinbrenner & Watson, 2015); thus, this increase in class size may have impacted student engagement between pretest and post-test.

In sum, there were no significant group differences on direct measures of student engagement and social-communication outcomes, and overall, there appeared to be limited change in the targeted outcomes from pre- to post-test. However, the video-anchored ratings showed that parents perceived growth in social-communication and play skills over the course of the study, which were driven by statistically significant changes in the ASAP-PD group.

### 4.3. Practical Factors

Despite the limited impact of ASAP-PD on implementation and student outcomes, both the ASAP-PD model and the ASAP intervention were regarded as feasible and useful by participating educators. Anecdotally, the educators often shared about the value of the ASAP skill hierarchies, which broke larger goals into smaller developmental steps. A large survey study of early childhood educators indicated that teachers’ belief in practices is related to their use of practices (Hugh et al., 2022), suggesting that educators’ positive perceptions of ASAP may be impactful on use of the intervention. Parents also had positive impressions of the ASAP intervention.

Although the educators had overall positive opinions about ASAP, educators did identify challenges related to their use of ASAP in the classroom. Educators noted external pressures that impacted their use of ASAP, including class and caseload size, continued additions of students throughout the year, and competing demands such as IEP meetings, school duties, and inconsistencies in staffing. These identified pressures are consistent with other research noting that major barriers to EBP implementation in preschools and schools serving autistic children include resources, time (for preparation, training, and planning), staffing issues (buy-in, staffing challenges), lack of training, and inconsistent use of EBPs across settings (see Barry et al., 2020 for a scoping review). The contextual factors of school settings deserve particular attention when considering intervention implementation, as there are important system-wide factors that influence educator implementation and student outcomes, including implementation climate, educator attitudes toward EBPs, and available resources (Stahmer et al., 2018; Suhrheinrich et al., 2021; Wilson & Landa, 2019).

### 4.4. Implications for Practice

Given that school districts are serving an increasing number of autistic students (Office of Special Education, 2025; Shaw et al., 2025) and educator shortages and turnover continue to be a challenge in special education (Gilmour et al., 2025; Learning Policy Institute, 2025), it is critical to understand how we can best support classroom teams in implementing EBPs in the current educational context.

One potential consideration is looking at classroom readiness. In this study, there was a lot of variability in general classroom practices across participating classrooms. It seems critical that classrooms have basic practices (e.g., structure, routines) and foundational EBPs (e.g., reinforcement, prompting, modeling) in place at the classroom level to facilitate use of any educational interventions. Anecdotally, we often saw visual evidence of EBPs but did not observe team members enacting use of the practices; examples include the presence of individual or classroom schedules in the classroom but minimal or inconsistent use of these tools during the school day. Thus, it may be important to assess classrooms and provide broader support as needed to put these foundational practices into place in a given classroom. Classroom-wide assessments such as the Early Childhood Environment Rating Scale-Third Edition (ECERS-3™, Harms et al., 2015) or Autism Program Environment Rating Scale (APERS^®^, Odom et al., 2024) may be useful in these efforts. Districts could utilize coaches to identify and address areas of need for individual classrooms. This obviously requires sufficient resources, which is a challenge in an era when educational funding is uncertain.

Another important consideration is team experience and capacity. One somewhat unique aspect of the ASAP-PD model is that it intentionally includes the full educational team—teachers, teaching assistants, and related service providers—in training and coaching. While this creates some unique challenges (e.g., need for dedicated time for PD activities, coordination of schedules across team members), it also provides advantages and is considered to be best practice for serving autistic children (Kunze & Machalicek, 2022). For example, it eases the burden of a single team member disseminating information to the broader team and allows for shared responsibility of implementation, thereby empowering multiple team members. Additionally, team-based interventions allow classrooms to take advantage of team members’ unique strengths, expertise, and experiences (Bateman et al., 2022). This may be particularly valuable in ASAP as it targets social-communication and play, intervention areas that would benefit from the inter-professional expertise of special educators, speech-language pathologists, and occupational therapists. It is important to note that differences in team experience and capacity may have implications for implementation. For example, a team with lower capacity may need more directed supporting for distributing and organizing the work of ASAP (i.e., cognitive participation in Normalization Process Theory (NPT, May et al., 2009), or teams with more limited experience with young autistic children may need additional training or coaching to embed and use EBPs to address ASAP goals (i.e., collective action in NPT). Additionally, the initial proposed ASAP-PD framework included establishing a cross-team community of practice to support educators’ use of ASAP. The small sample size and recruitment across school districts posed challenges to initiate this aspect of the PD framework in the current study. Future adaptations involving a larger sample size may utilize communities of practice to enhance the collective action (or operational work) of ASAP.

Finally, it seems important to help educators see the value of social-communication interventions and maintain motivation even when faced with challenges that are often outside of their control (e.g., class size, new students throughout the year, staffing challenges). Our overarching project used NPT (May et al., 2009) to guide our adaptation of ASAP-PD in earlier phases of the study. The coherence (or sense-making) aspect of NPT speaks to how teams buy into the intervention and map it onto existing practices. While not as explicitly addressed in the CRCT, our final ASAP-PD model embedded opportunities to link ASAP to student needs (e.g., expanding communication modalities, increasing initiations), classroom needs (e.g., embedded opportunities for communication across the classroom), as well as current classroom practices through self- and team-reflections built into the training modules and coaching meetings. However, the self-reports of ASAP use indicated that team members were often discouraged by these external factors, and this was evident anecdotally, as well as through coaching interactions. Thus, it is likely important to address educators’ perceptions of their locus of control in implementing EBPs. One such intervention is the Beliefs and Attitudes for Successful Implementation in Schools (BASIS), a pre-implementation support that has successfully supported educators in adoption of EBPs even in the midst of external challenges that exist in school settings (e.g., Cook et al., 2015; Lyon et al., 2019).

### 4.5. Future Directions

The adapted ASAP-PD model was still not sufficient in supporting optimal fidelity of ASAP in public preschool settings across all educational teams. The evidence of positive student outcomes from previous ASAP trials (Dykstra et al., 2012; Dykstra Steinbrenner et al., 2015; Watson et al., 2009), combined with the fact that the implementation challenges present in the current study are similar to other school-based interventions for autistic children (e.g., Barry et al., 2020; Wilson & Landa, 2019) suggest that examining implementation supports beyond enhanced PD may be warranted.

Although some of the quantitative data from the parallel IPE study was explored as part of this study, our research team is currently examining the qualitative data gathered from educational teams and our coaches in depth to identify key implementation barriers and challenges, which are pointing to structural barriers such as staffing and workload. This qualitative data can provide valuable information about gaps and challenges in current policies that impact educators’ capacity to implement interventions with fidelity. Using this existing data to explore potential implementation strategies that would address key barriers and challenges and then testing identified strategies in an implementation study will be an important next step for the ASAP intervention. Based on our data and experiences to date, implementation supports such as more intensive instructional coaching could help provide additional accountability. Additionally, providing temporary supplemental staffing during the initiation of implementation may help teams establish practices or routines to support integration and adoption of ASAP in the classroom. Given that communication is a core area of need and is considered a pivotal skill, supporting schools in identifying cost-effective implementation supports for communication interventions like ASAP is important for maximizing support for autistic students. Once these supports are identified and the anticipated level of impact is clearer, next steps could include conducting larger studies that are sufficiently powered based on anticipated effect sizes and examining classroom and/or educator-level moderators.

Beyond implementation, it also seems important to identify feasible measurement strategies to capture change in social-communication for autistic students. Although the recently adapted ECI-A is promising (Nowell et al., 2024), it was primarily designed for use with familiar play partners. Thus, the use of this tool with unfamiliar adults warrants further study. Additionally, since recruitment is a frequent challenge in school studies, there is an ongoing need for assessments that can be conducted remotely so that research teams are not as geographically limited in recruitment. The NIH Toolbox (Toolbox Assessments, Inc., 2026) has several social measures that have been validated for early childhood, so examining the use of those tools in school-based intervention studies may also be useful. Finally, it is possible that children with different characteristics at baseline may differ in developmental trajectories. In smaller studies when there are significant differences between children in groups (like this study), this can be problematic. While the statistical issues would likely be addressed by recruiting larger samples (i.e., great likelihood of similarity in groups with larger samples), it may also be helpful to consider more frequent measurement of skills to be able to look at child trajectories rather than only pre/post assessments.

### 4.6. Limitations

There are several limitations for this study. First, the pilot study was designed to be underpowered, but we recruited even fewer classrooms than planned, which may have further limited our ability to detect significant outcomes. The small sample size may impact the generalizability of findings. Second, odd numbers of schools in each year resulted in an uneven allocation to the ASAP-PD and ASAP-MO groups. Additionally, the groups ended up with significantly different mean Mullen-VR scores (a potential proxy for non-verbal developmental ability). Third, we also extended recruitment windows based on challenges with recruitment, which resulted in shorter timeframes for ASAP implementation. Of note, the trainings concluded just before winter break for four of the seven classrooms and occurred after winter break for three of the seven ASAP-PD classrooms; manuals were distributed after winter break for two of five ASAP-MO classrooms. Recruitment challenges also impacted the timelines for data collection for the second cohort. To minimize burden on these classroom teachers, we did not conduct the mid-point FOI interviews, as their timeline for intervention was already condensed. The lack of mid-point data collection for the interview portion of the FOI measure limited our ability to examine implementation fidelity change over the course of the school year. Fourth, although our ASAP FOI measures were adapted from previous ASAP trials, the measures incorporated changes based on previous experiences and recommendations from our advisory board (i.e., adjusting all items to a simplified 0–2 scale, adding a General Classroom Practices scale).

## 5. Conclusions

The adapted ASAP-PD model was successful in increasing educators’ knowledge and reported use of ASAP procedures compared to teams who only received access to ASAP materials, but it did not result in differences in observed FOI or student outcomes of engagement or communication. Notably, however, parents in the ASAP-PD group, but not those in the ASAP-MO group, perceived positive changes in their children’s social-communication skills on the video-anchored rating scale. Educators rated the acceptability of ASAP-PD and ASAP positively but noted external challenges in implementation. Future studies should examine implementation supports for effective use of EBPs to address social-communication with autistic preschool students. Policymakers should also consider external factors that impact the successful implementation of EBPs with young autistic students.

## Figures and Tables

**Table 1:** Educator Demographics by Group Assignment. This table includes number of educator participants and percentage of educator participants for demographic and descriptive variables including race/ethnicity, role, experience with autistic children, and highest level of education. Participants could select more than one race/ethnicity category; therefore, total percentages may exceed 100%.

	ASAP-MO (*n* = 15)		ASAP-PD (*n* = 26)	
Variable	*N*	%	*N*	%
***Race/Ethnicity***				
Asian	1	6.7	0	0.0
Black/African American	1	6.7	7	26.9
Hispanic/Latino/a	1	6.7	2	7.7
White	12	80.0	18	69.2
***Role***				
Lead teacher	5	33.3	7	26.9
Teaching assistant	6	40.0	12	46.1
Speech-language pathologist	3	20.0	4	15.4
Occupational therapist	1	6.7	3	11.5
***Experience with Autistic Children***				
<1 year	0	0.0	1	3.8
1–4 years	5	33.3	8	30.8
5–9 years	3	20.0	6	23.1
10+ years	7	46.7	11	42.3
***Highest Level of Education***				
High school/GED equivalency	0	0.0	1	3.8
Associate’s degree/Some college	3	20.0	7	26.9
Bachelor’s degree	5	33.3	9	34.6
Graduate degree	7	46.7	9	34.6

Note: ASAP = Advancing Social-Communication and Play intervention; ASAP-MO = group that received ASAP materials only; ASAP-PD = group that received ASAP professional development model; GED = general education development.

**Table 2:** Student Demographics by Group Assignment. This table includes number of participants and percentage of student participants for demographic variables including sex, race/ethnicity, and household income. Participants could select more than one race/ethnicity category; therefore, total percentages may exceed 100%.

	ASAP-MO (*n* = 18)		ASAP-PD (*n* = 25)	
Variable	*N*	%	*N*	%
***Sex at birth***				
Male	11	61.1	19	76.0
Female	7	38.9	6	24.0
***Race/Ethnicity***				
Asian	3	16.7	2	8.0
Black/African American	9	50.0	14	56.0
Hispanic/Latino/a	1	5.6	3	12.0
White	6	33.3	11	44.0
No response	1	5.6	0	0.0
***Household Income***				
<USD 20,000	2	11.1	2	8.0
USD 20,000–USD 39,999	2	11.1	7	28.0
USD 40,000–USD 59,999	2	11.1	6	24.0
USD 60,000–USD 79,999	4	22.2	3	12.0
USD 80,000–USD 99,999	0	0.0	1	4.0
≥USD 100,000	7	38.9	5	20.0
No response	1	5.6	1	4.0

Note: ASAP = Advancing Social-Communication and Play intervention; ASAP-MO = group that received ASAP materials only; ASAP-PD = group that received ASAP professional development model.

**Table 3:** ASAP FOI and General Classroom Practices Measures. This table describes fidelity features, item descriptions, and number of items in areas for ASAP Fidelity of Implementation and General Classroom Practices measures.

Fidelity Feature	Item Description	Items (All Scored 0–2 Points)
***ASAP Knowledge and Procedures*** *(collected via interview with lead teacher)*		
Goal	Use of ASAP terminology (reference to hierarchy, description of goal) when describing selected child SC and Play goal	4 items (1 each for SC and Play for 2 students)
Assessment	Educator use of ASAP assessments, process of goal selection, description of data-based decision-making across team	3 items
Teaming	Description of team membership (ideally lead teacher, at least one assistant, and at least one RSP), frequency of scheduled team meetings, team creation of action items targeting ASAP goals in focused and generalized contexts, amount and format of team communication	6 items
***ASAP Classroom Implementation*** *(collected via classroom observation)*		
Preparation	Team member(s) were prepared for the ASAP activity (e.g., materials ready)	1 item per activity
Implementation	Targeting an ASAP SC or Play goal during the activity, providing opportunities for child to demonstrate goal, selection of appropriate materials and activities (for activity and for child interest), setting expectations for child engagement, providing adequate wait time and prompting, providing reinforcement, and adapting materials/activities as needed	9 items
Monitoring	Team member(s) recorded student data related to child goals	1 item
Responsiveness	Quality of child engagement in terms of active engagement during the activity, maintaining attention, and enjoyment of the activity	3 items
***General Classroom Practices*** *(collected via classroom observation)*		
Use of EBPs	Educator use of EBPs including positive feedback, presence and use of daily schedule and visual supports, use of reinforcers, use of prompt hierarchies	5 items
Facilitating features	Environmental features of the classroom (organization, safety), student active engagement in learning activities, educator management of challenging behaviors, preparation for transitions, educator expression of respect and warmth, educator presentation of directions to students in a variety of methods based on student needs	8 items

**Table 4:** Generalized Linear Model Results: Intent-to-Treat Analyses Fidelity Outcomes. The generalized linear model (GLM) results comparing Advancing Social-Communication and Play (ASAP) with professional development (ASAP-PD) to ASAP with manual only (ASAP-MO) for three fidelity measures noted across the columns. Results include beta value coefficient estimates and standardized errors, and statistically significant effects that are less than *p* = 0.05 are noted with asterisks. Additionally, adjusted means, standard deviations, and effect sizes are reported for both groups.

Fixed Effect	ASAP Knowledge and Procedures (Mid-Point)			ASAP Knowledge and Procedures (Post-Test)			ASAP Classroom Observation—Focused Practice (Post-Test)		
	***B***	***SE***	**CI**	***B***	***SE***	**CI**	***B***	***SE***	**CI**
Intercept	0.35 *	0.30	−0.37–1.07	0.38 *	0.21	−0.08–0.85	0.77 ***	0.16	0.40–1.13
ASAP-PD	1.00 *	0.41	0.03–1.96	0.87 *	0.27	0.27–1.48	0.35	0.21	−0.13–0.83
**Adjusted Means**									
	**M**	**SD**	**ES**	**M**	**SD**	**ES**	**M**	**SD**	**ES**
ASAP-MO	0.35	0.24	1.73	0.38	0.39	1.94	0.77	0.18	1.02
ASAP-PD	1.35	0.78		1.26	0.51		1.12	0.45	

Note: ASAP = Advancing Social-Communication and Play; ASAP-PD = Advancing Social-Communication and Play Professional Development; * *p* < 0.05; *** *p* < 0.001. CI = 95% Confidence Interval.

**Table 5:** Hierarchical Linear Model Results: Intent-to-Treat Analyses Child Outcomes. Hierarchical linear model (HLM) results comparing Advancing Social-Communication and Play (ASAP) with professional development (ASAP-PD) to ASAP with manual only (ASAP-MO) for five student outcome measures noted across the columns. The HLM nests students within classrooms and includes student-level covariates of the pretest score of the tested outcome measure, the Mullen Scales of Early Learning Visual Reception (Mullen-VR) raw score, and age. Results include beta value coefficient estimates and standardized errors, and statistically significant effects that are less than *p* = 0.05 are noted with asterisks.

	Engagement			ECI-A: Weighted Comm. Rate			ECI-A: Directed Communication			ECI-A: Initiation of Joint Attention			BOSSC		
Fixed Effect	*B*	*SE*	CI	*B*	*SE*	CI	*B*	*SE*	CI	*B*	*SE*	CI	*B*	*SE*	CI
Intercept	11.43 ***	1.26	8.64–14.23	7.86 ***	1.11	5.39–10.33	1.61 ***	0.15	1.28–1.94	0.17 **	0.06	0.05–0.30	34.96 ***	1.77	30.97–38.96
ASAP-PD	1.62	1.74	−2.00–5.24	0.33	1.55	−2.90–3.56	−0.20	0.21	−0.63–0.23	−0.09	0.08	−0.25–0.07	3.06	2.53	−2.22–8.34
Pretest score	0.22	0.18	−0.14–0.58	0.72 ***	0.10	0.51–0.92	0.58 **	0.16	0.25–0.92	0.19	0.21	−0.24–0.62	0.70 ***	0.14	0.40–0.98
Mullen-VR	0.12	0.12	−0.13–0.37	0.01	0.11	−0.21–0.23	0.03	0.01	0.00–0.06	0.01	0.01	0.00–0.02	−0.26	0.18	−0.64–0.12
Age	−2.01	1.30	−4.71–0.68	1.26	1.12	−1.06–3.58	−0.01	0.09	−0.34–0.31	−0.05	0.06	−0.17–0.07	2.02	1.94	−2.02–6.07

Note: ASAP-PD = Advancing Social-Communication and Play Professional Development; ECI-A = Early Communication Indicator-Autism; BOSCC = Brief Observation of Social Communication Change; Comm. = Communication; VR = Visual Reception; ** *p* < 0.01; *** *p* < 0.001. CI = 95% Confidence Interval.

**Table 6:** Descriptive Statistics and Group Comparisons of Social Validity Measures. This table includes data from four measures related to social validity of the ASAP-PD and ASAP interventions and two measures related to the social validity of student outcomes separated by group assignment.

	ASAP-MO		ASAP-PD		Group Diff.	
**Social Validity-Interventions**	**Mean** **(SD)**	**Range**	**Mean** **(SD)**	**Range**	***p***	**ES**
PD-ASAP Training Rating 1–6 scale	NC	NC	5.03 (1.12)	1.40–6.00	--	--
CTA-Educator 1–5 scale	NC	NC	4.54 (0.31)	3.93–4.93	--	--
IRP Educator 1–6 scale	5.32 (0.70)	3.80–6.00	5.29 (0.52)	4.13–6.00	0.91	0.03
IRP Parent 1–6 scale	4.81 (0.58)	4.25–5.88	5.13 (0.68)	4.13–6.00	0.36	0.51
**Social Validity-Outcomes**	**Pre**	**Post**	**Pre**	**Post**	***p***	**ES**
VARS Educator 1–10 scale	2.55 (1.71)	3.58 (2.34)	3.04 (2.17)	3.21 (1.61)	0.45	0.25
VARS Parent 1–10 scale	3.17 (2.04)	5.11 (2.98)	2.98 (2.19)	4.11 (2.46)	0.81	0.09

Note: ASAP = Advancing Social-Communication and Play intervention; ASAP-MO = group that received ASAP materials only; ASAP-PD = group that received ASAP professional development model; CTA = Coach–Teacher Alliance; ES = effect size, and refers to the Absolute Standardized Mean Difference, or ASMD; IRP = Intervention Rating Profile; VARS = Video-Anchored Rating Scale; NC = not collected, -- = not calculated.

## Data Availability

The datasets generated and/or analyzed during the current study are available from the corresponding author on reasonable request.

## Supplementary Materials

The following supporting information can be downloaded at: https://www.mdpi.com/article/10.3390/bs16081363/s1, Table S1: Baseline Characteristics of Analytic Sample by Condition; Table S2: Attrition Analyses Comparing Students Who Completed and Withdrew from the Study.
