# Supplementary material for: Examining the Impact of an Adapted Professional Development Model for the Advancing Social-Communication and Play (ASAP) Intervention
Source: Behav Sci (Basel). 2026 Aug 9;16(8):1363. doi: 10.3390/bs16081363 (PMC13509528; doi:10.3390/bs16081363)
Supplement: Supplementary file 1 [file behavsci-16-01363-s001.zip › behavsci-4411569-supplementary.pdf]

Table S1.

Baseline Characteristics of Analytic Sample by Condition (n = 43)

	ASAP-MO			ASAP-PD			Group diff.	
	N	Prop./ Mean	SD	N	Prop./ Mean	SD	*p*	*ES*
Age (years)	18	4.43	0.61	25	4.30	0.58	.47	0.22
Gender (male)	18	0.61	--	25	0.76	--	.29	0.33
Household Income	18	4.28	1.93	25	3.52	1.78	.19	0.41
Mullen-VR	17	27.35	6.52	24	22.88	7.49	**.05**	**0.64**
Engagement	18	12.64	4.39	25	12.50	4.38	.92	0.03
ECI-A: Weighted Communication Rate	18	9.50	9.35	25	7.11	5.87	.35	0.31
ECI-A: Directed Communication	18	1.31	0.59	25	1.13	0.66	.37	0.29
ECI-A: Initiation of Joint Attention	18	0.15	0.19	25	0.10	0.18	.36	0.27
BOSCC	18	35.67	9.89	25	37.60	10.15	.54	0.19

Note: ASAP = Advancing Social-communication And Play intervention; ASAP-MO = group that received ASAP materials only; ASAP-PD = group that received ASAP professional development model; BOSCC = Brief Observation of Social Communication Change; Diff. = differences; ECI-A = Early Communication Indicator – Autism; ES = effect size; Prop. = proportion; VR = Visual Reception

Table S2.

Attrition Analyses Comparing Students Who Completed and Withdrew from the Study (N=43)

	Completed Intervention			Withdrew from Study			Group diff.	
Variable	N	Prop./ Mean	SD	N	Prop./ Mean	SD	*p*	*ES*
Age (years)	39	4.43	0.62	4	4.48	0.20	.66	0.30
Gender (male)	39	0.72	--	4	0.50	--	.37	0.47
Household Income	39	3.82	1.78	4	4.00	2.94	.86	0.07
Mullen-VR	37	24.11	7.04	4	30.50	8.89	.10	0.80
Engagement	39	12.35	4.47	4	14.63	1.70	.32	0.67
ECI-A: Weighted Communication Rate	39	7.49	6.98	4	14.17	10.98	.09	0.73
ECI-A: Directed Communication	39	1.17	0.63	4	1.50	0.54	.33	0.56
ECI-A: Initiation of Joint Attention	39	0.12	0.19	4	0.13	0.14	.97	0.06
BOSCC	39	37.32	9.10	4	31.63	17.23	.56	0.41

Note: BOSCC = Brief Observation of Social Communication Change; Diff. = differences; ECI-A = Early Communication Indicator – Autism; ES = effect size; Prop. = proportion; VR = Visual Reception
